# Supplementary material for: Gut opportunistic pathogens contribute to high-altitude pulmonary edema by elevating lysophosphatidylcholines and inducing inflammation
Source: Microbiol Spectr. 2025 May 22;13(7):e03057-24. doi: 10.1128/spectrum.03057-24 (PMC12210943; doi:10.1128/spectrum.03057-24)
Supplement: Supplemental material — Fig. S1 to S9; Tables S1 and S2. [file spectrum.03057-24-s0001.pdf]

Supporting information for

Gut opportunistic pathogens contribute to high altitude pulmonary edema by elevating lysophosphatidylcholines and inducing inflammation

Xianduo Sun[1], Gaosheng Hu[1], Yuting Li[2], Wenjing Li[1], Yong Wang[1], Hui Yan[1], Guoqing Long[1], Long Zhao[1], Anhua Wang[1,**] and Jingming Jia[1,*]

[1]Department of Traditional Chinese Materia Medica, Shenyang Pharmaceutical University, Shenyang 110016, China.

[2]Department of Pharmacology, Shenyang Pharmaceutical University, Shenyang, 110016, China.

Corresponding authors

[*]Corresponding author: jiajingming@syphu.edu.cn (J. Jia)

[**]Corresponding author: sywanganhua@163.com (A. Wang)

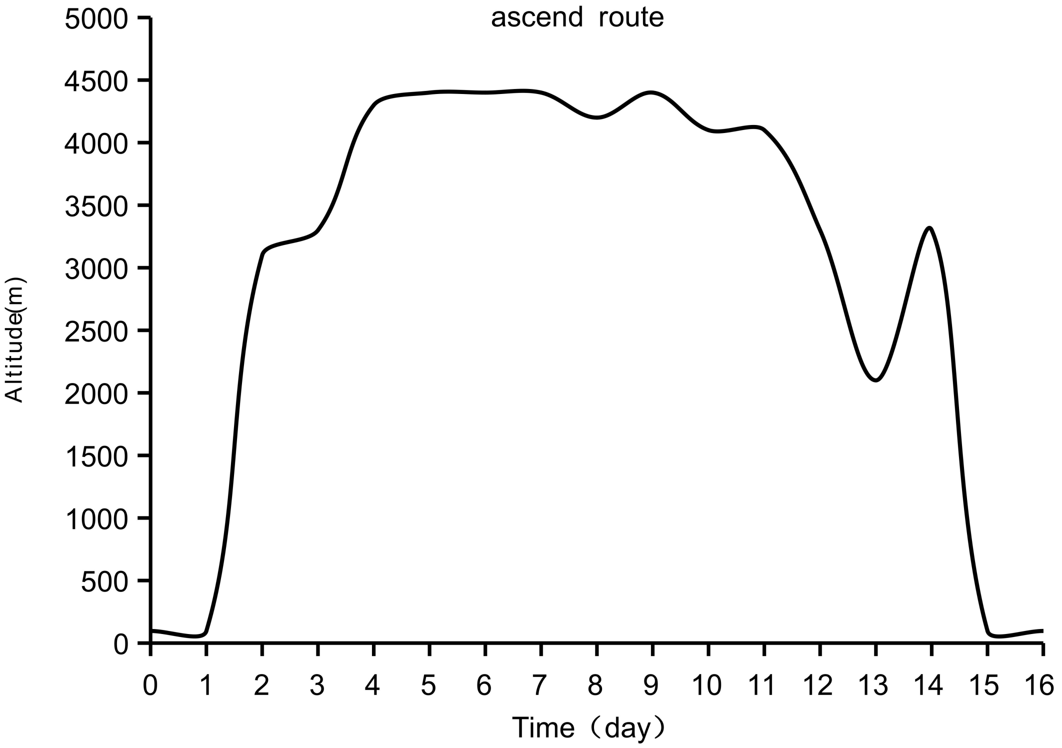

Fig. S1. Ascent profile.

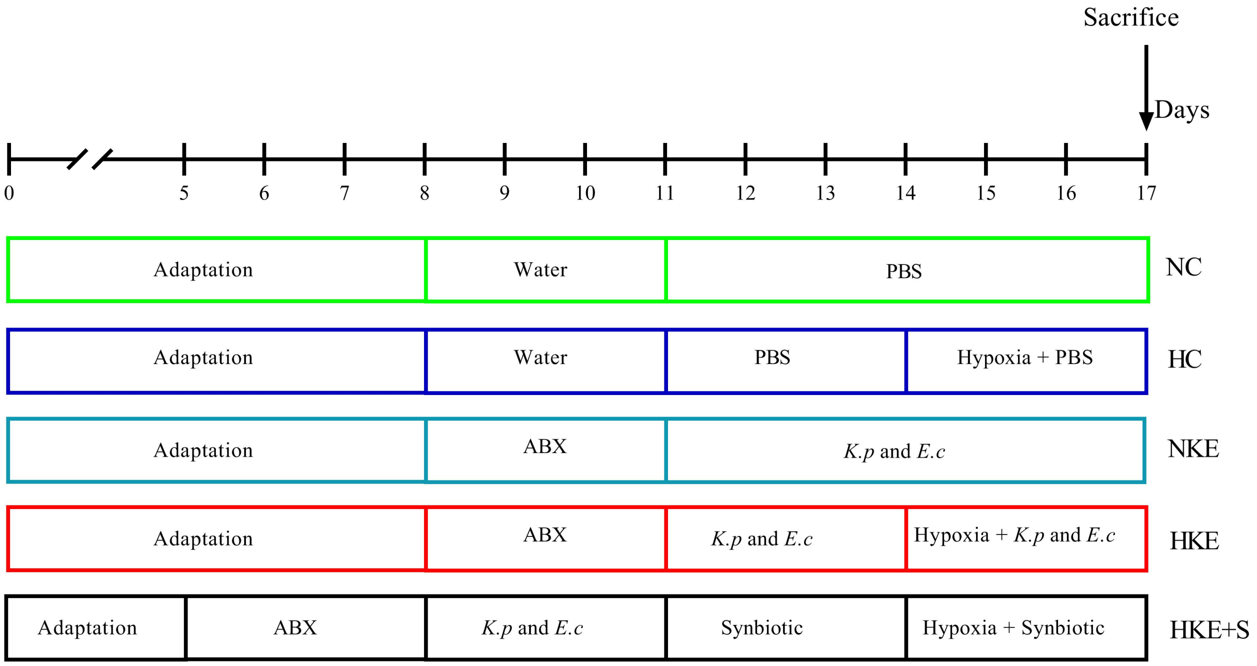

Fig. S2. Experimental timeline.

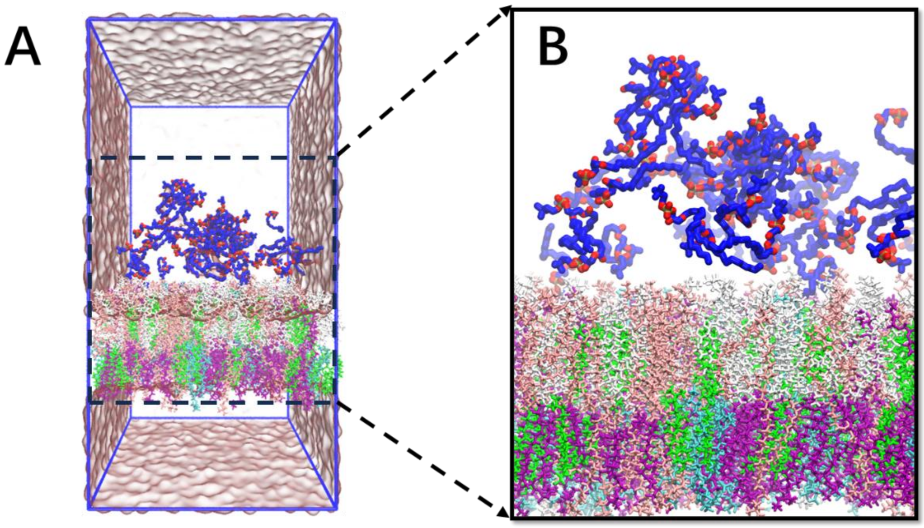

Fig. S3. The initial configuration of the simulation system. A shows that the cell membrane is composed of several lipids represented by different colours: green stick model for CHL1, white for PSM, pink for POPC, cyan for POPS and purple for POPE. LPC molecules are shown in blue and are randomly distributed in the upper part of the membrane. The water molecules are shown in a cotton-like transparent form surrounding the outside of the entire system, but the transparency is reduced in this figure to better visualise the internal structure. Panel B is an enlarged view focusing on the detailed arrangement between the LPC molecules and the cell membrane lipids, which provides important structural information for studying the interaction between them.

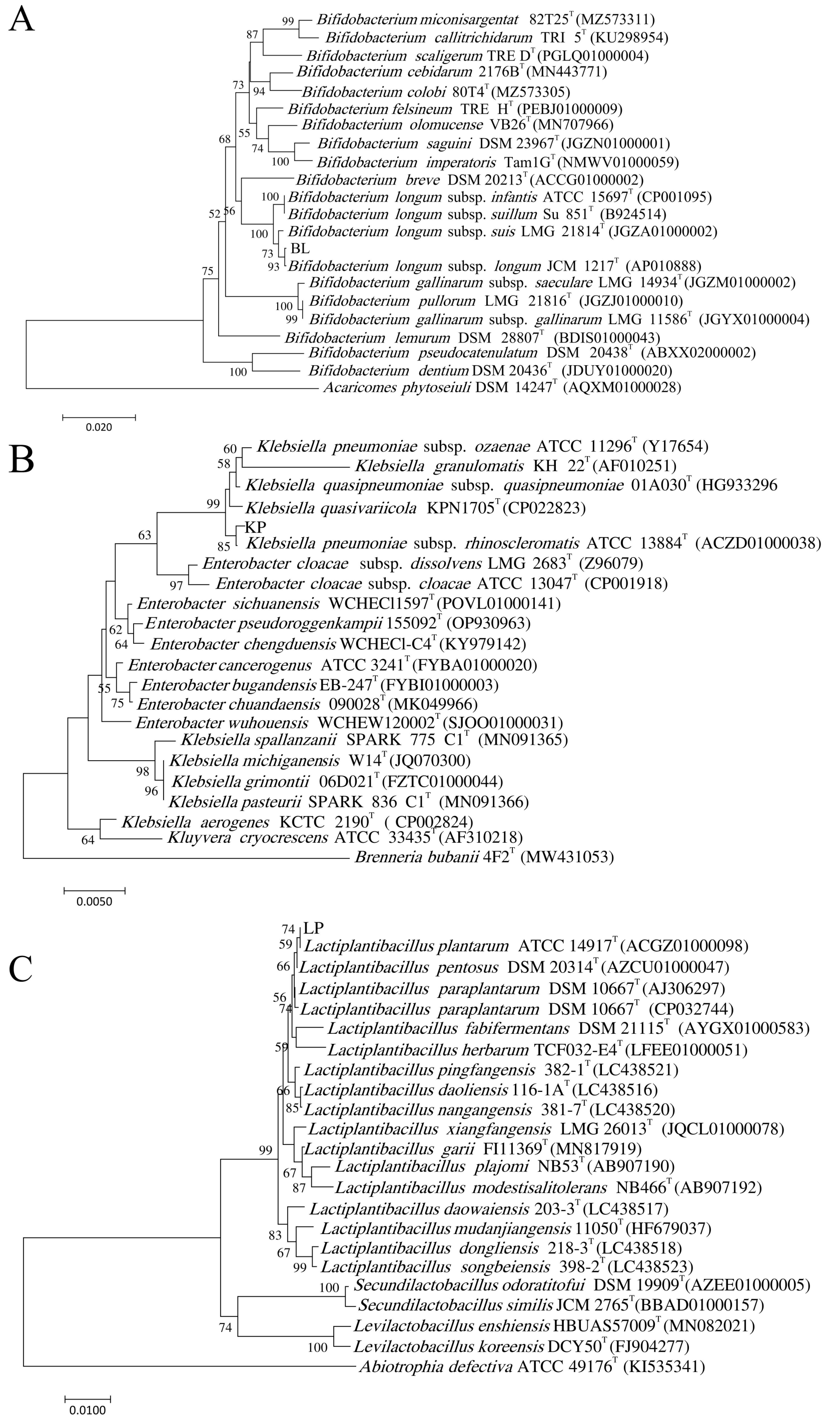

Fig. S4. Phylogenetic tree for 16S rRNA of BL, KP and LP strains using the NJ method based on the maximum likelihood method. (A) Phylogenetic tree for 16S rRNA of BL strains. (B) Phylogenetic tree for 16S rRNA of KP strains. (C) Phylogenetic tree for 16S rRNA of LP strains. Two strains isolated from fresh fecal samples of the HA group were designated as KP and EC, two strains isolated from fresh fecal samples of the HR group were designated as BP and BL, and one strain isolated from fresh fecal samples of the HG group was designated as LP. The 16S rRNA gene sequence of BP and EC showed 100% similarity to *Bifidobacterium pseudocatenulatum* (MH719034.1) and *Escherichia coli* (CP010134.1), respectively.

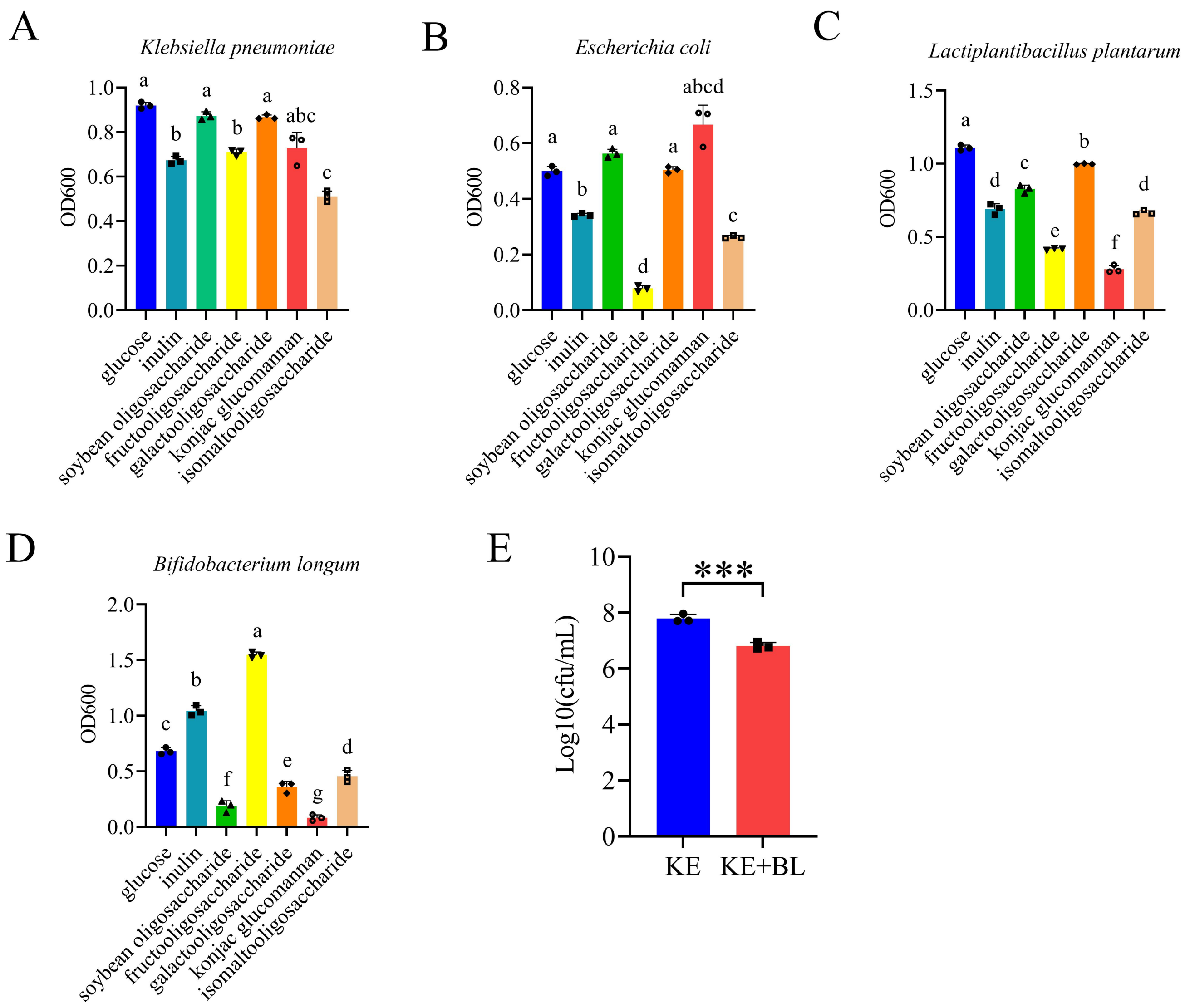

Fig. S5. The optimal prebiotics for *B. longum* and *L. plantarum* and their combined inhibition of the growth of *E. coli* and *K. pneumoniae*. (A-D) Effects of prebiotics on the growth of *Klebsiella pneumoniae* (A), *Escherichia coli* (B), *Lactiplantibacillus plantarum* (C) and *Bifidobacterium longum* (D) (n = 3). Statistical analysis was performed by one-way ANOVA. Different letters indicate significant difference (A-D). Data are means ± SD. (E) Effect of *L. plantarum* and *B. longum* on log colony forming units (CFU)/mL of *K. pneumoniae* and *E. coli* (n = 3). Statistical analysis was performed using Student's t-test (E). ***$P < 0.001$. Data are means ± SD.

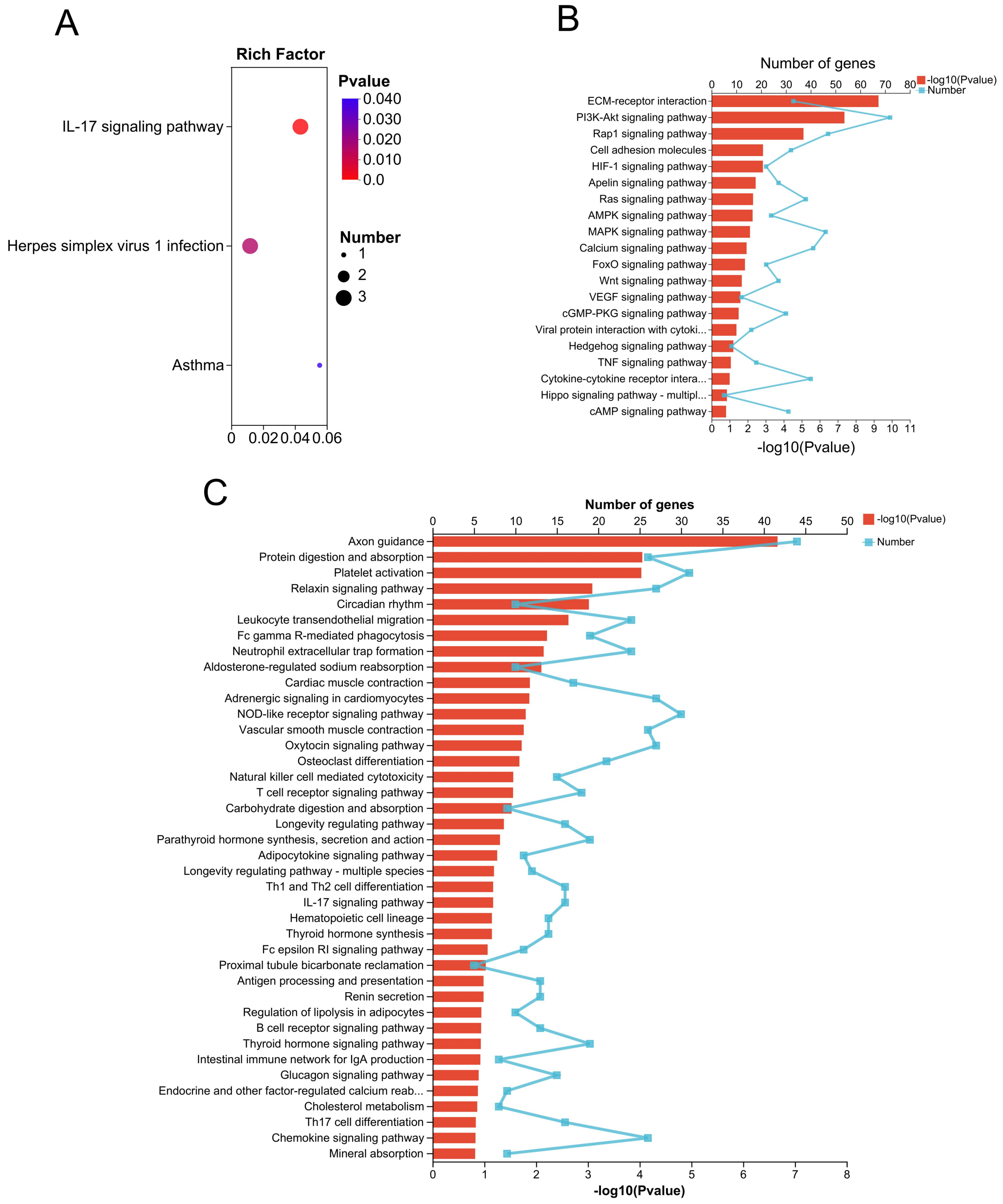

Fig. S6. KEGG pathway enrichment analysis. (A) Bubble plot of KEGG pathway enrichment analysis of differentially expressed genes between HKE and HKE+S groups. (B, C) Bar graph of KEGG pathway enrichment analysis of differentially expressed genes between HKE and NC groups.

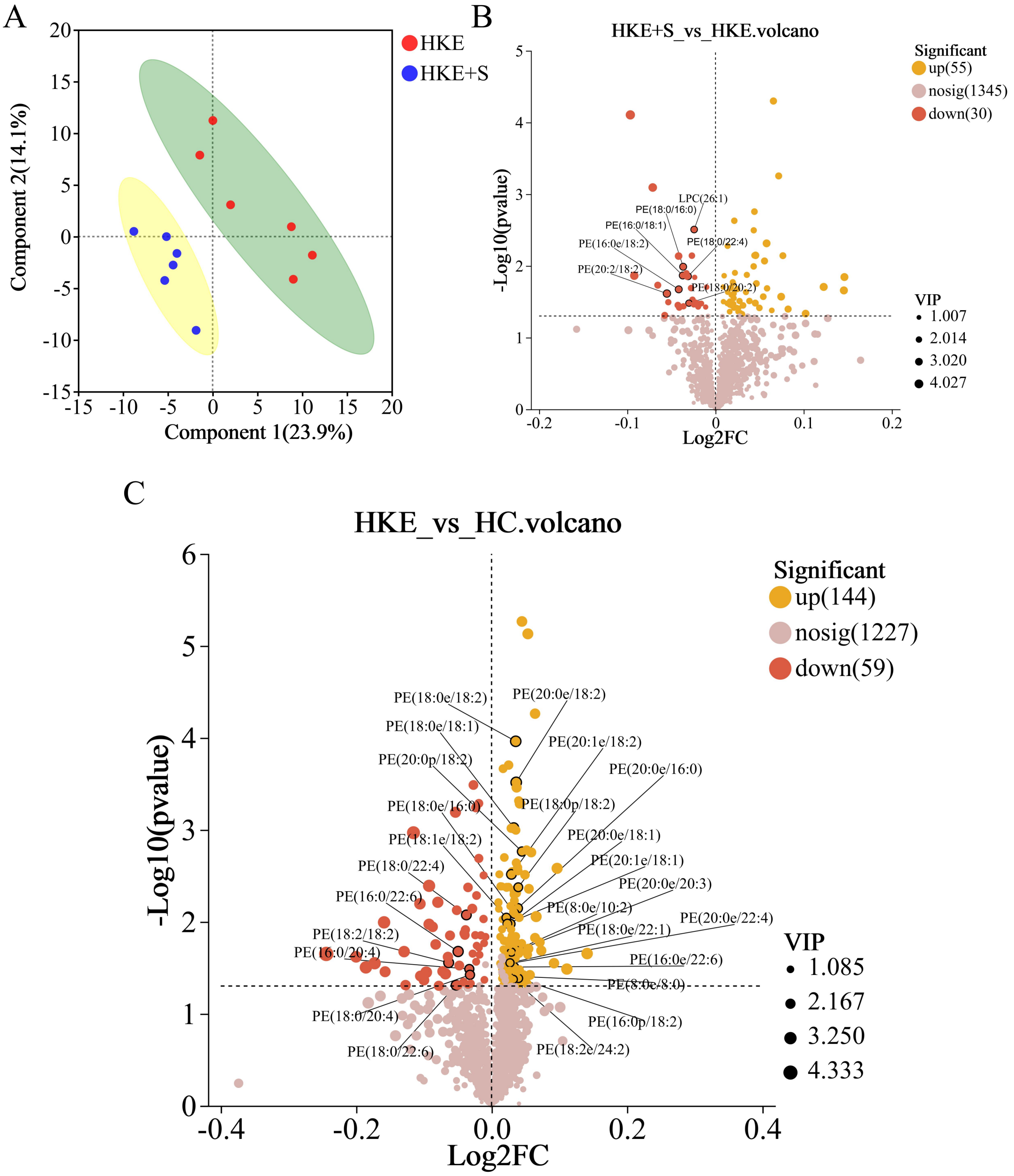

Fig. S7. *E.coli* and *K. pneumoniae* disrupt lipid metabolism under hypoxic conditions. (A) Partial least squares discriminant analysis between HKE and HKE+S groups. (B, C) Volcano plot of differential metabolites between HKE+S and HKE (B), as well as between HKE and HC (C).

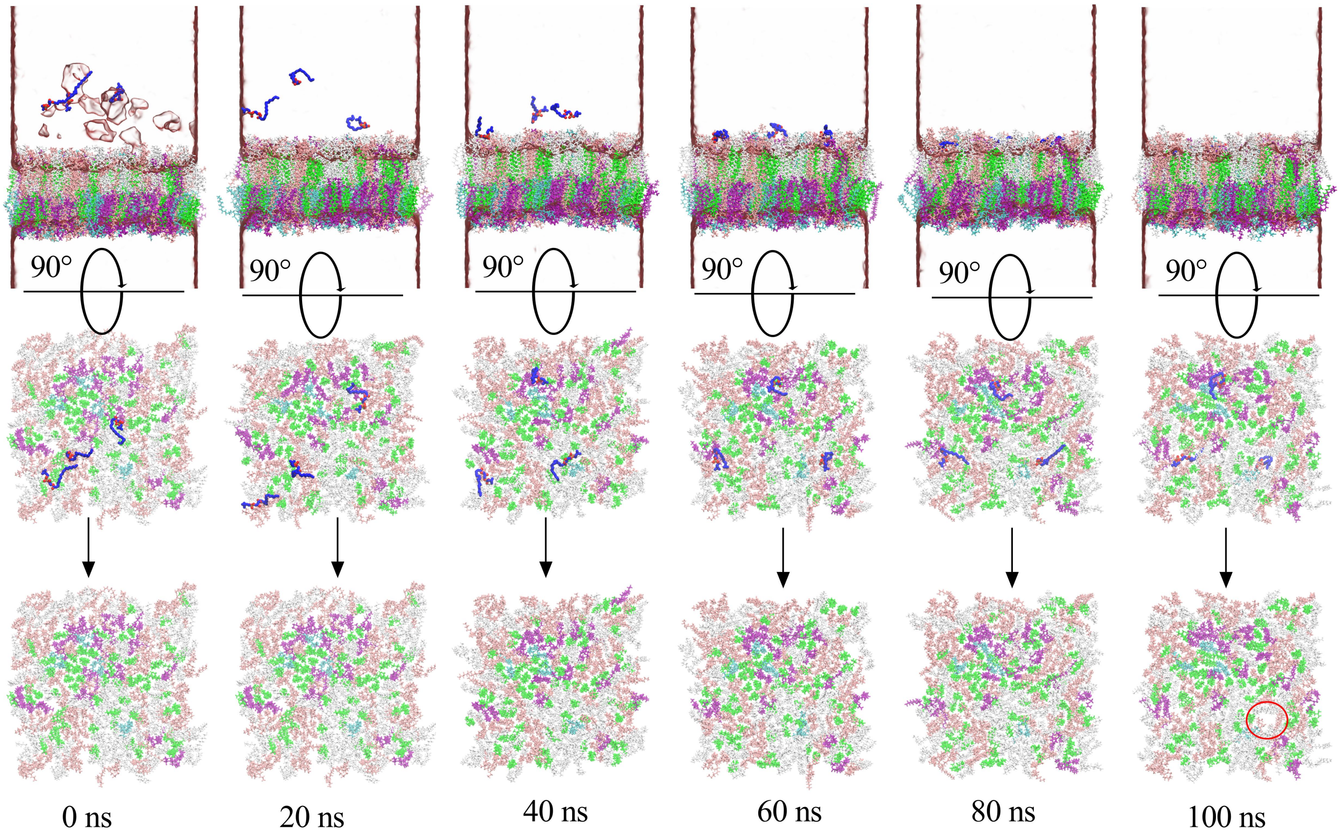

Fig. S8. The molecular dynamics simulation of the interaction process between LPCs of 6.25 μg/mL and cell membranes. The cell membrane is composed of a variety of lipids represented by different colours: green for CHL1, white for PSM, pink for POPC, cyan for POPS and purple for POPE. LPC molecules are shown in blue and are randomly distributed in the upper part of the membrane. Water molecules are shown in a cotton-like transparent form that surrounds the outside of the entire system.

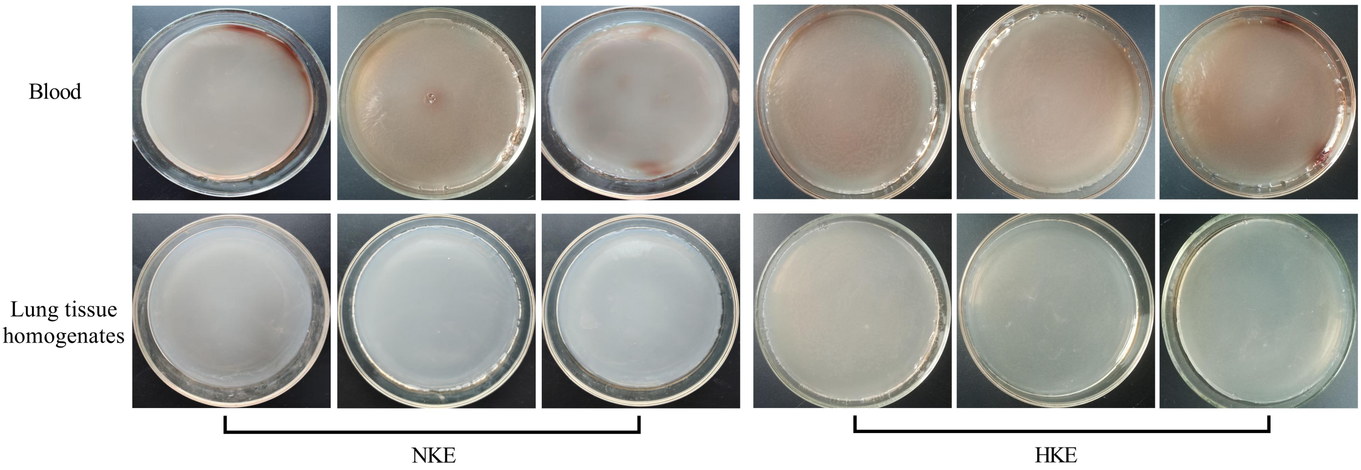

Fig. S9. Incubation of blood and lung tissue homogenates from rats in the NKE and HKE groups (approximately 0.1 g of tissue dissolved in 2 mL of sterile PBS) with basal salt medium containing 1% glucose at 37 °C for 3 days.

Tab. S1. The 2018 Lake Louise Acute Mountain Sickness Score.

volunteers	Scoring items	day 0	day 1	day 2	day 3	day 4	day 5	day 6	day 7	day 8	day 9	day 10	day 11	day 12	day 13	day 14	day 15
VM01	Headache	0	0	1	1	2	2	1	1	1	0	0	0	0	0	0	0
	Gastrointestinal	0	0	0	0	1	0	1	0	0	0	0	0	0	0	0	0
	Fatigue and/or	0	0	1	1	2	1	1	1	1	0	1	0	0	0	0	0
	Dizziness/light-head	0	0	2	2	1	2	1	1	1	0	0	0	0	0	0	0
	Total score	0△	0	4	4	6▽	5	4	3	3	0	1◇	0	0	0	0	0
VM02	Headache	0	0	1	1	2	2	1	1	1	1	0	0	0	0	0	0
	Gastrointestinal	0	0	1	1	1	1	1	1	0	1	1	1	0	0	0	0
	Fatigue and/or	0	0	1	1	2	1	1	1	1	0	1	0	0	0	0	0
	Dizziness/light-head	0	0	1	1	1	1	0	0	0	0	0	0	0	0	0	0
	Total score	0△	0	4	4	6▽	5	3	3	2	2	2◇	1	0	0	0	0
VM03	Headache	0	0	1	1	2	3	3	1	1	1	1	0	0	0	0	0
	Gastrointestinal	0	0	0	0	0	0	1	0	0	0	0	0	0	0	0	0
	Fatigue and/or	0	0	1	1	2	3	3	1	1	1	1	1	0	0	0	0
	Dizziness/light-head	0	0	1	1	2	2	2	1	1	1	0	0	0	0	0	0
	Total score	0△	0	3	3	6	8	9▽	3	3	3	2◇	1	0	0	0	0
VM04	Headache	0	0	0	1	2	2	1	1	1	0	0	0	0	0	0	0
	Gastrointestinal	0	0	0	1	1	1	1	1	1	0	0	0	0	0	0	0
	Fatigue and/or	0	0	0	1	1	1	1	0	0	0	0	0	0	0	0	0
	Dizziness/light-head	0	0	0	1	1	1	1	1	1	0	0	0	0	0	0	0
	Total score	0△	0	0	4	5▽	5	4	3	3	0◇	0	0	0	0	0	0
VM05	Headache	0	0	2	1	1	2	1	2	1	1	0	0	0	0	0	0
	Gastrointestinal	0	0	0	0	0	0	0	1	1	0	0	0	0	0	0	0
	Fatigue and/or	0	0	0	1	1	1	2	1	1	2	1	1	0	0	0	0
	Dizziness/light-head	0	0	1	1	2	1	1	2	1	1	1	0	0	0	0	0
	Total score	0△	0	3	3	4	4▽	4	6	4	4	2◇	1	0	0	0	0
VM06	Headache	0	0	0	1	2	2	1	1	1	0	0	0	0	0	0	0
	Gastrointestinal	0	0	0	0	1	1	2	2	1	1	1	0	0	0	0	0
	Fatigue and/or	0	0	1	1	0	0	0	0	0	0	1	1	1	0	0	0
	Dizziness/light-head	0	0	1	1	0	0	0	0	0	0	0	0	0	0	0	0
	Total score	0△	0	2	3	3▽	3	3	3	2	1	2◇	1	1	0	0	0
VM07	Headache	0	0	1	1	2	2	1	0	0	0	0	0	0	0	0	0
	Gastrointestinal	0	0	1	0	0	0	0	0	0	0	0	0	0	0	0	0
	Fatigue and/or	0	0	1	0	0	0	0	0	0	0	0	0	0	0	0	0
	Dizziness/light-head	0	0	1	1	1	1	0	0	1	0	0	0	0	0	0	0
	Total score	0△	0	4▽	2	3	3	1	0	1	0	0◇	0	0	0	0	0
VM08	Headache	0	0	1	2	2	2	2	0	1	0	0	0	0	0	0	0
	Gastrointestinal	0	0	0	0	0	0	0	0	0	0	0	0	0	0	0	0
	Fatigue and/or	0	0	1	1	1	1	1	0	0	0	0	0	0	0	0	0
	Dizziness/light-head	0	0	1	2	2	2	2	1	1	1	1	0	0	0	0	0
	Total score	0△	0	3	5	5	5▽	5	1	2	1	1◇	0	0	0	0	0
VM09	Headache	0	0	0	0	0	1	1	0	0	0	0	0	0	0	0	0
	Gastrointestinal	0	0	0	0	0	0	0	0	0	0	0	0	0	0	0	0
	Fatigue and/or	0	0	0	0	0	1	1	1	1	0	0	0	0	0	0	0
	Dizziness/light-head	0	0	0	0	0	0	0	0	0	0	0	0	0	0	0	0
	Total score	0	0	0	0	0	2	2	1	1	0	0	0	0	0	0	0
VM10	Headache	0	0	1	1	3	3	2	1	1	1	0	0	0	0	0	0
	Gastrointestinal	0	0	0	2	1	2	1	1	1	1	1	0	0	0	0	0
	Fatigue and/or	0	0	1	3	1	2	1	1	1	1	1	1	1	1	0	0
	Dizziness/light-head	0	0	1	1	2	3	1	1	1	1	1	1	0	0	0	0
	Total score	0△	0	3	7	7▽	10	5	4	4	4	3◇	2	1	1	0	0
VW11	Headache	0	0	0	0	2	2	1	0	0	0	0	0	0	0	0	0
	Gastrointestinal	0	0	1	1	1	1	0	0	0	0	0	0	0	0	0	0
	Fatigue and/or	0	0	0	1	2	2	1	0	0	1	0	0	0	0	0	0
	Dizziness/light-head	0	0	0	0	2	2	0	0	0	0	0	0	0	0	0	0
	Total score	0△	0	1	2	7▽	7	2	0	0	1◇	0	0	0	0	0	0
VW12	Headache	0	0	1	2	2	2	1	1	0	0	0	0	0	0	0	0
	Gastrointestinal	0	0	2	1	0	0	0	0	0	0	0	0	0	0	0	0
	Fatigue and/or	0	0	2	1	1	1	1	1	0	1	1	0	0	0	0	0
	Dizziness/light-head	0	0	1	0	0	0	0	0	1	0	0	0	0	0	0	0
	Total score	0△	0	6▽	4	3	3	2	2	1	1	1◇	0	0	0	0	0
VW13	Headache	0	0	0	1	2	2	2	1	1	1	1	0	0	0	0	0
	Gastrointestinal	0	0	0	1	1	1	1	1	0	0	0	0	0	0	0	0
	Fatigue and/or	0	0	0	1	2	2	2	1	1	0	0	0	0	0	0	0
	Dizziness/light-head	0	0	1	1	2	2	2	1	1	1	0	0	0	0	0	0
	Total score	0△	0	1	4	7▽	7	7	4	3	2	1◇	0	0	0	0	0
VM14	Headache	0	0	0	0	0	1	0	0	0	0	0	0	0	0	0	0
	Gastrointestinal	0	0	0	0	0	0	1	0	0	0	0	0	0	0	0	0
	Fatigue and/or	0	0	0	1	0	0	0	0	0	0	0	0	0	0	0	0
	Dizziness/light-head	0	0	0	0	0	0	0	0	0	0	0	0	0	0	0	0
	Total score	0	0	0	1	0	1	1	0	0	0	0	0	0	0	0	0
VM15	Headache	0	0	0	1	1	1	1	1	1	0	0	0	0	0	0	0
	Gastrointestinal	0	0	0	1	1	0	0	0	0	0	0	1	0	0	0	0
	Fatigue and/or	0	0	1	1	2	2	2	1	1	1	1	0	0	0	0	0
	Dizziness/light-head	0	0	1	1	2	2	1	1	1	1	0	0	0	0	0	0
	Total score	0△	0	2	4	6▽	5	4	3	3	2	1◇	1	0	0	0	0

Table s1 (continued). The 2018 Lake Louise Acute Mountain Sickness Score.

volunteers	Scoring items	day 0	day 1	day 2	day 3	day 4	day 5	day 6	day 7	day 8	day 9	day 10	day 11	day 12	day 13	day 14	day 15
	Headache	0	0	0	1	1	1	1	1	1	0	0	0	0	0	0	0
	Gastrointestinal	0	0	1	1	1	1	1	0	1	1	1	0	0	0	0	0
VW16	Fatigue and/or	0	0	0	0	1	2	1	1	0	1	1	1	0	0	0	0
	Dizziness/light-head	0	0	1	1	1	1	1	0	0	0	0	0	0	0	0	0
	Total score	0△	0	2	3	4	5▽	4	2	2	2	2◇	1	0	0	0	0
	Headache	0	0	0	1	1	0	0	0	0	0	0	0	0	0	0	0
	Gastrointestinal	0	0	1	1	1	1	1	0	0	0	0	0	0	0	0	0
VW17	Fatigue and/or	0	1	1	0	0	0	0	1	0	0	0	0	0	0	0	0
	Dizziness/light-head	0	0	0	0	0	1	0	0	0	0	0	0	0	0	0	0
	Total score	0	1	2	2	2	2	1	1	0	0	0	0	0	0	0	0
	Headache	0	0	0	0	1	0	0	0	0	0	0	0	0	0	0	0
	Gastrointestinal	0	0	1	1	1	0	1	0	0	0	0	0	0	0	0	0
VW18	Fatigue and/or	0	0	0	1	0	1	0	1	0	1	0	0	0	0	0	0
	Dizziness/light-head	0	0	0	0	0	0	0	1	0	0	0	0	0	0	0	0
	Total score	0	0	1	2	2	1	1	2	0	1	0	0	0	0	0	0
	Headache	0	0	1	1	2	1	1	1	1	0	0	0	0	0	0	0
	Gastrointestinal	0	0	1	1	2	1	1	1	1	0	0	0	0	0	0	0
VW19	Fatigue and/or	0	0	1	1	1	1	1	1	1	1	0	0	0	0	0	0
	Dizziness/light-head	0	0	0	0	1	1	0	0	0	0	0	0	0	0	0	0
	Total score	0△	0	3	3	6▽	4	3	3	3	1	0◇	0	0	0	0	0
	Headache	0	0	1	1	1	1	0	0	1	0	0	0	0	0	0	0
	Gastrointestinal	0	0	2	1	1	1	1	1	1	1	0	1	0	0	0	0
VW20	Fatigue and/or	0	0	1	1	1	1	1	1	1	0	0	0	0	0	0	0
	Dizziness/light-head	0	0	1	1	1	0	0	0	0	0	0	0	0	0	0	0
	Total score	0△	0	5▽	4	4	3	2	2	3	1	0◇	1	0	0	0	0
	Headache	0	0	0	1	1	0	0	0	0	0	0	0	0	0	0	0
	Gastrointestinal	0	0	0	1	0	1	0	1	0	1	0	0	0	0	0	0
VW21	Fatigue and/or	0	0	1	0	1	1	1	0	0	0	0	0	0	0	0	0
	Dizziness/light-head	0	0	0	0	0	0	0	0	0	0	0	0	0	0	0	0
	Total score	0	0	1	2	2	2	1	1	0	1	0	0	0	0	0	0
	Headache	0	0	1	1	2	1	1	0	1	0	0	0	0	0	0	0
	Gastrointestinal	0	0	1	1	1	1	1	1	1	1	0	0	0	0	0	0
VW22	Fatigue and/or	0	0	1	1	1	1	1	1	1	1	0	0	0	0	0	0
	Dizziness/light-head	0	0	0	0	1	1	0	1	0	0	0	0	0	0	0	0
	Total score	0△	0	3	3	5▽	4	3	3	3	2	0	0	0	0	0	0
	Headache	0	0	0	1	1	1	1	1	1	0	0	0	0	0	0	0
	Gastrointestinal	0	1	1	0	1	1	0	1	1	0	0	0	0	0	0	0
VW23	Fatigue and/or	0	0	0	1	1	1	1	1	0	1	0	0	0	0	0	0
	Dizziness/light-head	0	0	0	0	0	1	1	0	0	0	0	0	0	0	0	0
	Total score	0△	1	1	2	3	4▽	3	3	2	1	0◇	0	0	0	0	0
	Headache	0	0	1	1	1	1	1	1	0	0	0	0	0	0	0	0
	Gastrointestinal	0	0	0	1	1	0	1	0	1	0	1	0	0	0	0	0
VW24	Fatigue and/or	0	1	1	1	1	1	1	1	1	1	0	0	0	0	0	0
	Dizziness/light-head	0	0	0	0	1	1	1	0	0	0	0	0	0	0	0	0
	Total score	0△	1	2	3	4▽	3	4	2	2	1	1	0	0	0	0	0
	Headache	0	0	1	1	2	1	1	1	1	0	0	0	0	0	0	0
	Gastrointestinal	0	0	0	1	1	1	1	1	1	1	0	0	0	0	0	0
VM25	Fatigue and/or	0	0	1	1	1	2	1	1	1	1	1	1	0	0	0	0
	Dizziness/light-head	0	0	0	0	1	0	1	0	0	0	0	0	0	0	0	0
	Total score	0△	0	2	3	5▽	4	4	3	3	2	1◇	1	0	0	0	0
	Headache	0	0	1	1	1	1	1	1	0	0	0	0	0	0	0	0
	Gastrointestinal	0	0	0	0	1	1	1	1	1	0	1	0	0	0	0	0
VM26	Fatigue and/or	0	0	0	1	2	2	1	1	1	1	0	1	0	0	0	0
	Dizziness/light-head	0	0	0	0	0	1	0	0	0	0	0	0	0	0	0	0
	Total score	0△	0	1	2	4	5▽	3	3	2	1	1◇	1	0	0	0	0
	Headache	0	0	1	1	2	2	1	1	0	0	0	0	0	0	0	0
	Gastrointestinal	0	0	1	1	0	1	1	0	0	0	0	0	0	0	0	0
VM27	Fatigue and/or	0	0	1	1	2	1	2	2	1	1	0	0	0	0	0	0
	Dizziness/light-head	0	0	0	0	0	1	1	0	0	0	0	0	0	0	0	0
	Total score	0△	0	3	3	4	5▽	5	3	1	1	0◇	0	0	0	0	0
	Headache	0	0	1	1	2	1	1	1	1	1	0	0	0	0	0	0
	Gastrointestinal	0	0	1	1	1	1	1	1	1	0	0	0	0	0	0	0
VM28	Fatigue and/or	0	0	1	1	2	2	1	1	1	1	1	1	1	0	0	0
	Dizziness/light-head	0	0	0	0	1	1	1	0	0	0	0	0	0	0	0	0
	Total score	0△	0	3	3	6▽	5	4	3	3	2	1◇	1	1	0	0	0
	Headache	0	0	0	1	1	1	1	1	1	0	0	0	0	0	0	0
	Gastrointestinal	0	0	1	1	1	0	1	0	1	0	0	1	0	0	0	0
VM29	Fatigue and/or	0	0	1	1	1	1	0	1	1	1	0	0	0	0	0	0
	Dizziness/light-head	0	0	0	0	1	0	0	0	0	0	0	0	0	0	0	0
	Total score	0△	0	2	3	4▽	2	2	2	3	1	0	1	0	0	0	0
	Headache	0	0	0	1	0	0	0	0	0	0	0	0	0	0	0	0
	Gastrointestinal	0	0	0	1	1	0	0	0	0	0	0	0	0	0	0	0
VM30	Fatigue and/or	0	0	1	0	1	1	0	0	0	0	0	0	0	0	0	0
	Dizziness/light-head	0	0	0	0	0	0	0	0	0	0	0	0	0	0	0	0
	Total score	0	0	1	2	2	1	0	0	0	0	0	0	0	0	0	0

Tab. S2. The composition and number of molecules in the two simulated systems.

Components	100 ug/mL LPCs	6.25 ug/mL LPCs
LPC	50	3
CHL1	136	136
PSM	63	63
POPC	73	73
POPS	45	45
POPE	83	83
SOD	236	236
CLA	191	191
TIP3 water	55204	55204
